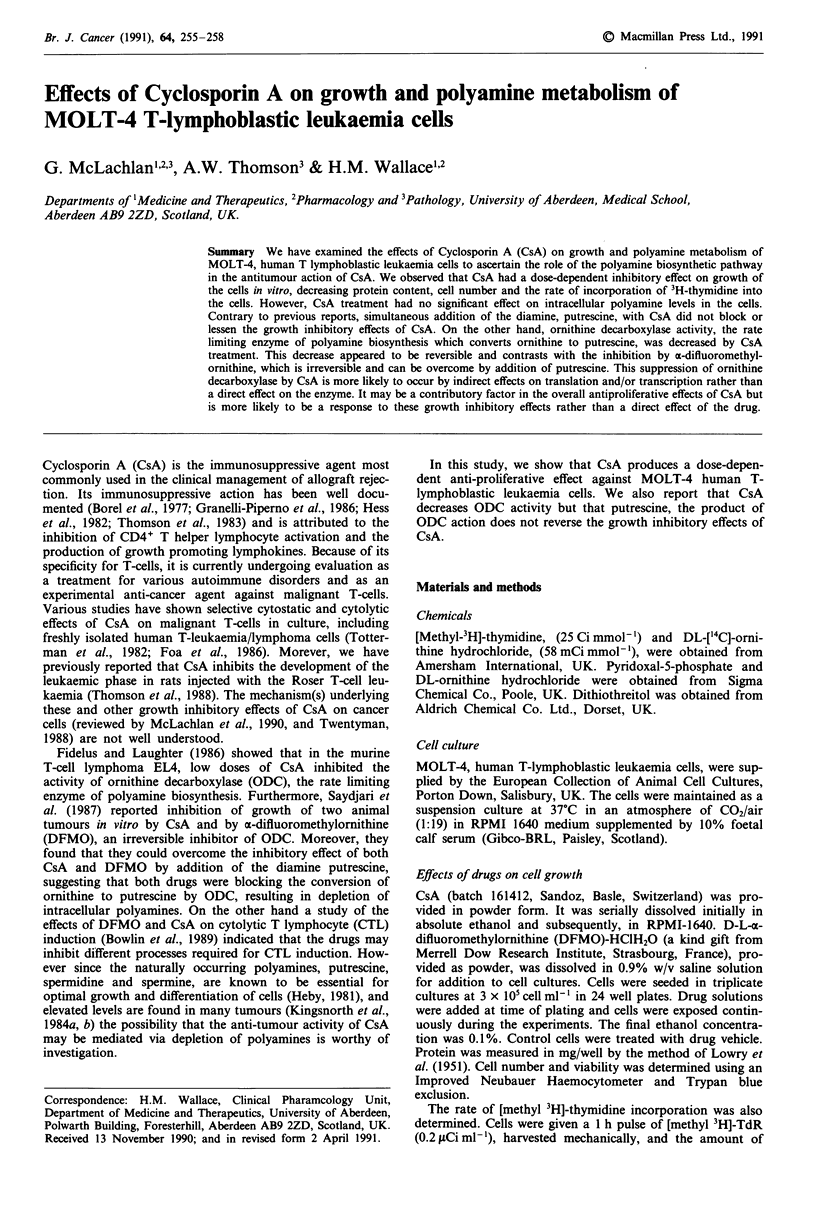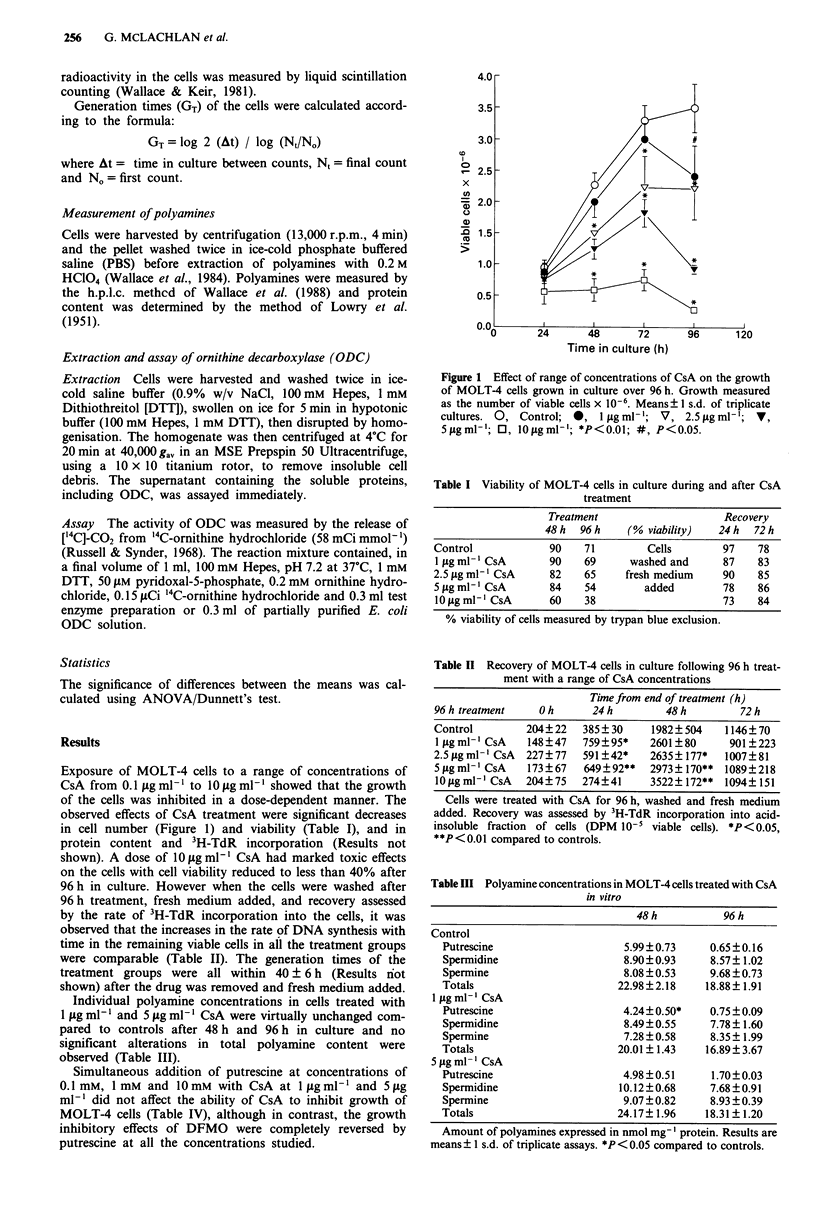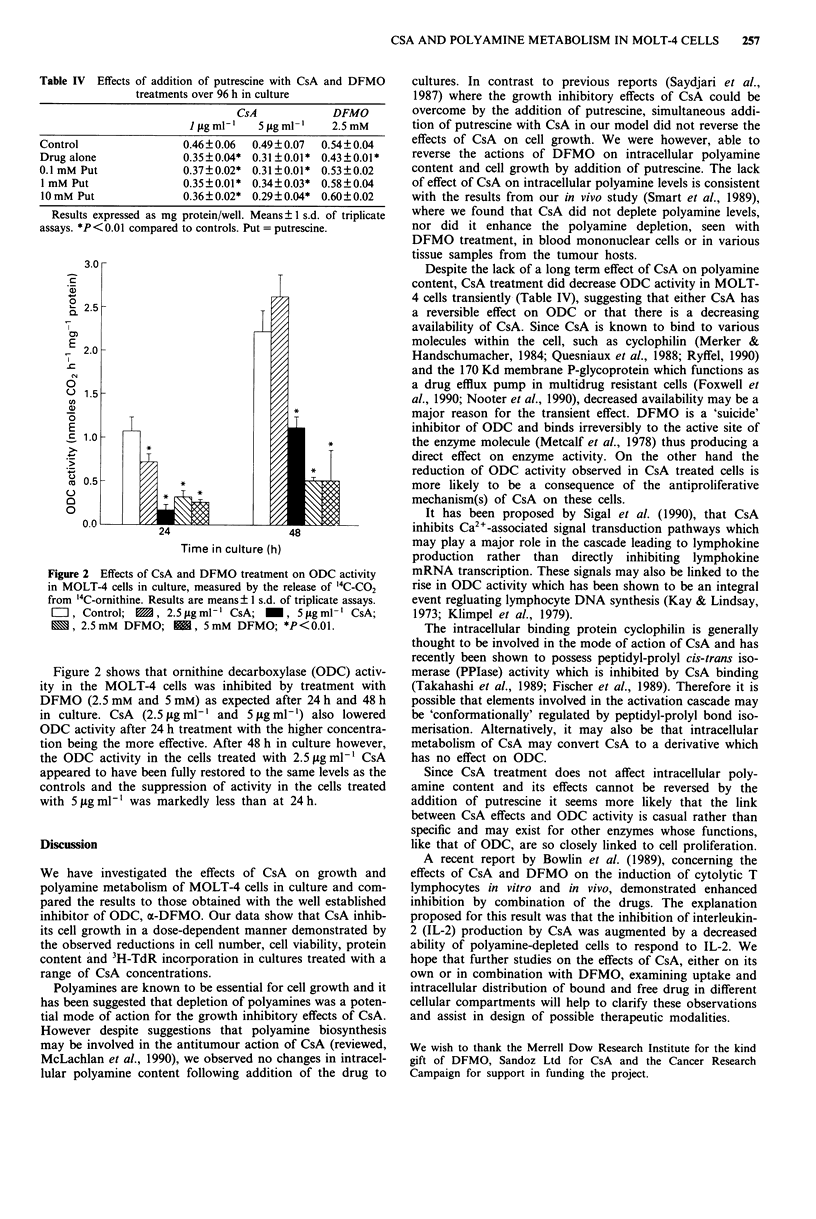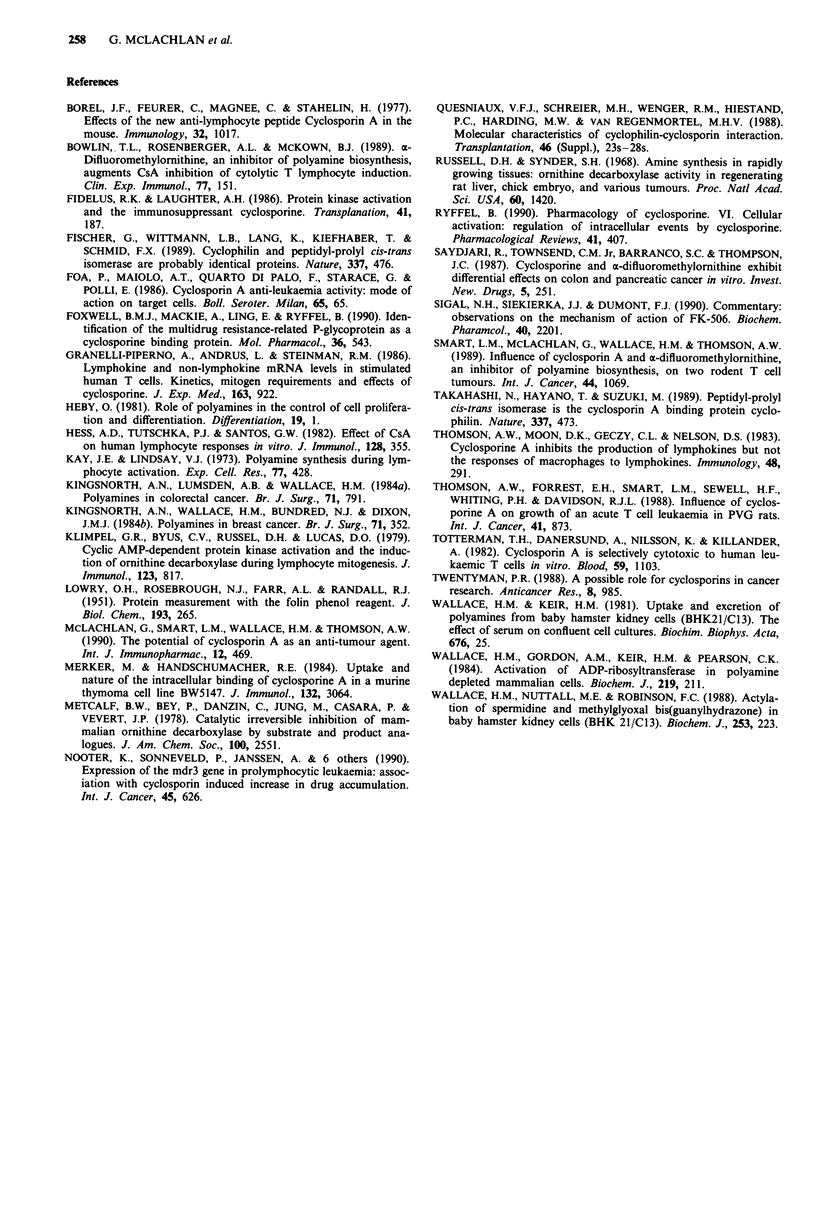# Effects of cyclosporin A on growth and polyamine metabolism of MOLT-4 T-lymphoblastic leukaemia cells.

**DOI:** 10.1038/bjc.1991.287

**Published:** 1991-08

**Authors:** G. McLachlan, A. W. Thomson, H. M. Wallace

**Affiliations:** Department of Medicine, University of Aberdeen, Medical School, Scotland, UK.

## Abstract

We have examined the effects of Cyclosporin A (CsA) on growth and polyamine metabolism of MOLT-4, human T lymphoblastic leukaemia cells to ascertain the role of the polyamine biosynthetic pathway in the antitumour action of CsA. We observed that CsA had a dose-dependent inhibitory effect on growth of the cells in vitro, decreasing protein content, cell number and the rate of incorporation of 3H-thymidine into the cells. However, CsA treatment had no significant effect on intracellular polyamine levels in the cells. Contrary to previous reports, simultaneous addition of the diamine, putrescine, with CsA did not block or lessen the growth inhibitory effects of CsA. On the other hand, ornithine decarboxylase activity, the rate limiting enzyme of polyamine biosynthesis which converts ornithine to putrescine, was decreased by CsA treatment. This decrease appeared to be reversible and contrasts with the inhibition by alpha-difluoromethyl-ornithine, which is irreversible and can be overcome by addition of putrescine. This suppression of ornithine decarboxylase by CsA is more likely to occur by indirect effects on translation and/or transcription rather than a direct effect on the enzyme. It may be a contributory factor in the overall antiproliferative effects of CsA but is more likely to be a response to these growth inhibitory effects rather than a direct effect of the drug.


Br. J. Cancer (1991), 64, 255-258 © Macmillan Press Ltd., 1991

### Effects of Cyclosporin A on growth and polyamine metabolism of MOLT-4 T-lymphoblastic leukaemia cells

G. McLachlan[1,2,3], A.W. Thomson[3] & H.M. Wallace[1,2]

*Departments of [1]Medicine and Therapeutics, [2]Pharmacology and [3]Pathology, University of Aberdeen, Medical School, Aberdeen AB9 2ZD, Scotland, UK.*

**Summary** We have examined the effects of Cyclosporin A (CsA) on growth and polyamine metabolism of MOLT-4, human T lymphoblastic leukaemia cells to ascertain the role of the polyamine biosynthetic pathway in the antitumour action of CsA. We observed that CsA had a dose-dependent inhibitory effect on growth of the cells *in vitro*, decreasing protein content, cell number and the rate of incorporation of $^{3}$H-thymidine into the cells. However, CsA treatment had no significant effect on intracellular polyamine levels in the cells. Contrary to previous reports, simultaneous addition of the diamine, putrescine, with CsA did not block or lessen the growth inhibitory effects of CsA. On the other hand, ornithine decarboxylase activity, the rate limiting enzyme of polyamine biosynthesis which converts ornithine to putrescine, was decreased by CsA treatment. This decrease appeared to be reversible and contrasts with the inhibition by α-difluoromethylornithine, which is irreversible and can be overcome by addition of putrescine. This suppression of ornithine decarboxylase by CsA is more likely to occur by indirect effects on translation and/or transcription rather than a direct effect on the enzyme. It may be a contributory factor in the overall antiproliferative effects of CsA but is more likely to be a response to these growth inhibitory effects rather than a direct effect of the drug.

Cyclosporin A (CsA) is the immunosuppressive agent most commonly used in the clinical management of allograft rejection. Its immunosuppressive action has been well documented (Borel *et al.*, 1977; Granelli-Piperno *et al.*, 1986; Hess *et al.*, 1982; Thomson *et al.*, 1983) and is attributed to the inhibition of $CD4^{+}$ T helper lymphocyte activation and the production of growth promoting lymphokines. Because of its specificity for T-cells, it is currently undergoing evaluation as a treatment for various autoimmune disorders and as an experimental anti-cancer agent against malignant T-cells. Various studies have shown selective cytostatic and cytolytic effects of CsA on malignant T-cells in culture, including freshly isolated human T-leukaemia/lymphoma cells (Totterman *et al.*, 1982; Foa *et al.*, 1986). Morever, we have previously reported that CsA inhibits the development of the leukaemic phase in rats injected with the Roser T-cell leukaemia (Thomson *et al.*, 1988). The mechanism(s) underlying these and other growth inhibitory effects of CsA on cancer cells (reviewed by McLachlan *et al.*, 1990, and Twentyman, 1988) are not well understood.

Fidelus and Laughter (1986) showed that in the murine T-cell lymphoma EL4, low doses of CsA inhibited the activity of ornithine decarboxylase (ODC), the rate limiting enzyme of polyamine biosynthesis. Furthermore, Saydjari *et al.* (1987) reported inhibition of growth of two animal tumours *in vitro* by CsA and by α-difluoromethylornithine (DFMO), an irreversible inhibitor of ODC. Moreover, they found that they could overcome the inhibitory effect of both CsA and DFMO by addition of the diamine putrescine, suggesting that both drugs were blocking the conversion of ornithine to putrescine by ODC, resulting in depletion of intracellular polyamines. On the other hand a study of the effects of DFMO and CsA on cytolytic T lymphocyte (CTL) induction (Bowlin *et al.*, 1989) indicated that the drugs may inhibit different processes required for CTL induction. However since the naturally occurring polyamines, putrescine, spermidine and spermine, are known to be essential for optimal growth and differentiation of cells (Heby, 1981), and elevated levels are found in many tumours (Kingsnorth *et al.*, 1984*a*, *b*) the possibility that the anti-tumour activity of CsA may be mediated via depletion of polyamines is worthy of investigation.

Correspondence: H.M. Wallace, Clinical Pharamcology Unit, Department of Medicine and Therapeutics, University of Aberdeen, Polwarth Building, Foresterhill, Aberdeen AB9 2ZD, Scotland, UK.
Received 13 November 1990; and in revised form 2 April 1991.

In this study, we show that CsA produces a dose-dependent anti-proliferative effect against MOLT-4 human T-lymphoblastic leukaemia cells. We also report that CsA decreases ODC activity but that putrescine, the product of ODC action does not reverse the growth inhibitory effects of CsA.

### Materials and methods

### *Chemicals*

[Methyl-$^{3}$H]-thymidine, (25 Ci $mmol^{-1}$) and DL-[$^{14}$C]-ornithine hydrochloride, (58 mCi $mmol^{-1}$), were obtained from Amersham International, UK. Pyridoxal-5-phosphate and DL-ornithine hydrochloride were obtained from Sigma Chemical Co., Poole, UK. Dithiothreitol was obtained from Aldrich Chemical Co. Ltd., Dorset, UK.

### *Cell culture*

MOLT-4, human T-lymphoblastic leukaemia cells, were supplied by the European Collection of Animal Cell Cultures, Porton Down, Salisbury, UK. The cells were maintained as a suspension culture at 37°C in an atmosphere of $CO_2$/air (1:19) in RPMI 1640 medium supplemented by 10% foetal calf serum (Gibco-BRL, Paisley, Scotland).

### *Effects of drugs on cell growth*

CsA (batch 161412, Sandoz, Basle, Switzerland) was provided in powder form. It was serially dissolved initially in absolute ethanol and subsequently, in RPMI-1640. D-L-α-difluoromethylornithine (DFMO)-$HClH_2O$ (a kind gift from Merrell Dow Research Institute, Strasbourg, France), provided as powder, was dissolved in 0.9% w/v saline solution for addition to cell cultures. Cells were seeded in triplicate cultures at $3 \times 10^{5}$ cell $ml^{-1}$ in 24 well plates. Drug solutions were added at time of plating and cells were exposed continuously during the experiments. The final ethanol concentration was 0.1%. Control cells were treated with drug vehicle. Protein was measured in mg/well by the method of Lowry *et al.* (1951). Cell number and viability was determined using an Improved Neubauer Haemocytometer and Trypan blue exclusion.

The rate of [methyl $^{3}$H]-thymidine incorporation was also determined. Cells were given a 1 h pulse of [methyl $^{3}$H]-TdR (0.2 μCi $ml^{-1}$), harvested mechanically, and the amount of radioactivity in the cells was measured by liquid scintillation counting (Wallace & Keir, 1981).

Generation times ($G_T$) of the cells were calculated according to the formula:

$$G_T = \log 2\ (\Delta t)\ /\ \log\ (N_t/N_o)$$

where $\Delta t$ = time in culture between counts, $N_t$ = final count and $N_o$ = first count.

*Measurement of polyamines*

Cells were harvested by centrifugation (13,000 r.p.m., 4 min) and the pellet washed twice in ice-cold phosphate buffered saline (PBS) before extraction of polyamines with 0.2 M $HClO_4$ (Wallace *et al.*, 1984). Polyamines were measured by the h.p.l.c. methcd of Wallace *et al.* (1988) and protein content was determined by the method of Lowry *et al.* (1951).

*Extraction and assay of ornithine decarboxylase (ODC)*

*Extraction* Cells were harvested and washed twice in ice-cold saline buffer (0.9% w/v NaCl, 100 mM Hepes, 1 mM Dithiothreitol [DTT]), swollen on ice for 5 min in hypotonic buffer (100 mM Hepes, 1 mM DTT), then disrupted by homogenisation. The homogenate was then centrifuged at 4°C for 20 min at 40,000 $g_{av}$ in an MSE Prepspin 50 Ultracentrifuge, using a 10 × 10 titanium rotor, to remove insoluble cell debris. The supernatant containing the soluble proteins, including ODC, was assayed immediately.

*Assay* The activity of ODC was measured by the release of $[^{14}C]$-$CO_2$ from $^{14}C$-ornithine hydrochloride (58 mCi $mmol^{-1}$) (Russell & Synder, 1968). The reaction mixture contained, in a final volume of 1 ml, 100 mM Hepes, pH 7.2 at 37°C, 1 mM DTT, 50 μM pyridoxal-5-phosphate, 0.2 mM ornithine hydrochloride, 0.15 μCi $^{14}C$-ornithine hydrochloride and 0.3 ml test enzyme preparation or 0.3 ml of partially purified *E. coli* ODC solution.

*Statistics*

The significance of differences between the means was calculated using ANOVA/Dunnett's test.

### Results

Exposure of MOLT-4 cells to a range of concentrations of CsA from 0.1 μg $ml^{-1}$ to 10 μg $ml^{-1}$ showed that the growth of the cells was inhibited in a dose-dependent manner. The observed effects of CsA treatment were significant decreases in cell number (Figure 1) and viability (Table I), and in protein content and $^3H$-TdR incorporation (Results not shown). A dose of 10 μg $ml^{-1}$ CsA had marked toxic effects on the cells with cell viability reduced to less than 40% after 96 h in culture. However when the cells were washed after 96 h treatment, fresh medium added, and recovery assessed by the rate of $^3H$-TdR incorporation into the cells, it was observed that the increases in the rate of DNA synthesis with time in the remaining viable cells in all the treatment groups were comparable (Table II). The generation times of the treatment groups were all within 40 ± 6 h (Results not shown) after the drug was removed and fresh medium added.

Individual polyamine concentrations in cells treated with 1 μg $ml^{-1}$ and 5 μg $ml^{-1}$ CsA were virtually unchanged compared to controls after 48 h and 96 h in culture and no significant alterations in total polyamine content were observed (Table III).

Simultaneous addition of putrescine at concentrations of 0.1 mM, 1 mM and 10 mM with CsA at 1 μg $ml^{-1}$ and 5 μg $ml^{-1}$ did not affect the ability of CsA to inhibit growth of MOLT-4 cells (Table IV), although in contrast, the growth inhibitory effects of DFMO were completely reversed by putrescine at all the concentrations studied.

**Figure 1** Effect of range of concentrations of CsA on the growth of MOLT-4 cells grown in culture over 96 h. Growth measured as the number of viable cells × $10^{-6}$. Means ± 1 s.d. of triplicate cultures. O, Control; ●, 1 μg $ml^{-1}$; ∇, 2.5 μg $ml^{-1}$; ▼, 5 μg $ml^{-1}$; □, 10 μg $ml^{-1}$; *$P<0.01$; #, $P<0.05$.

**Table I** Viability of MOLT-4 cells in culture during and after CsA treatment

| | *Treatment* 48 h | 96 h | (% viability) | *Recovery* 24 h | 72 h |
|---|---|---|---|---|---|
| Control | 90 | 71 | Cells | 97 | 78 |
| 1 μg $ml^{-1}$ CsA | 90 | 69 | washed and | 87 | 83 |
| 2.5 μg $ml^{-1}$ CsA | 82 | 65 | fresh medium | 90 | 85 |
| 5 μg $ml^{-1}$ CsA | 84 | 54 | added | 78 | 86 |
| 10 μg $ml^{-1}$ CsA | 60 | 38 | | 73 | 84 |

% viability of cells measured by trypan blue exclusion.

**Table II** Recovery of MOLT-4 cells in culture following 96 h treatment with a range of CsA concentrations

| *96 h treatment* | *Time from end of treatment (h)* 0 h | 24 h | 48 h | 72 h |
|---|---|---|---|---|
| Control | 204±22 | 385±30 | 1982±504 | 1146±70 |
| 1 μg $ml^{-1}$ CsA | 148±47 | 759±95* | 2601±80 | 901±223 |
| 2.5 μg $ml^{-1}$ CsA | 227±77 | 591±42* | 2635±177* | 1007±81 |
| 5 μg $ml^{-1}$ CsA | 173±67 | 649±92** | 2973±170** | 1089±218 |
| 10 μg $ml^{-1}$ CsA | 204±75 | 274±41 | 3522±172** | 1094±151 |

Cells were treated with CsA for 96 h, washed and fresh medium added. Recovery was assessed by $^3H$-TdR incorporation into acid-insoluble fraction of cells (DPM $10^{-5}$ viable cells). *$P<0.05$, **$P<0.01$ compared to controls.

**Table III** Polyamine concentrations in MOLT-4 cells treated with CsA *in vitro*

| | *48 h* | *96 h* |
|---|---|---|
| Control | | |
| Putrescine | 5.99±0.73 | 0.65±0.16 |
| Spermidine | 8.90±0.93 | 8.57±1.02 |
| Spermine | 8.08±0.53 | 9.68±0.73 |
| Totals | 22.98±2.18 | 18.88±1.91 |
| 1 μg $ml^{-1}$ CsA | | |
| Putrescine | 4.24±0.50* | 0.75±0.09 |
| Spermidine | 8.49±0.55 | 7.78±1.60 |
| Spermine | 7.28±0.58 | 8.35±1.99 |
| Totals | 20.01±1.43 | 16.89±3.67 |
| 5 μg $ml^{-1}$ CsA | | |
| Putrescine | 4.98±0.51 | 1.70±0.03 |
| Spermidine | 10.12±0.68 | 7.68±0.91 |
| Spermine | 9.07±0.82 | 8.93±0.39 |
| Totals | 24.17±1.96 | 18.31±1.20 |

Amount of polyamines expressed in nmol $mg^{-1}$ protein. Results are means ± 1 s.d. of triplicate assays. *$P<0.05$ compared to controls.

**Table IV** Effects of addition of putrescine with CsA and DFMO treatments over 96 h in culture

| | *CsA* | | *DFMO* |
|---|---|---|---|
| | *1* $\mu$g ml$^{-1}$ | 5 $\mu$g ml$^{-1}$ | 2.5 mM |
| Control | 0.46 ± 0.06 | 0.49 ± 0.07 | 0.54 ± 0.04 |
| Drug alone | 0.35 ± 0.04* | 0.31 ± 0.01* | 0.43 ± 0.01* |
| 0.1 mM Put | 0.37 ± 0.02* | 0.31 ± 0.01* | 0.53 ± 0.02 |
| 1 mM Put | 0.35 ± 0.01* | 0.34 ± 0.03* | 0.58 ± 0.04 |
| 10 mM Put | 0.36 ± 0.02* | 0.29 ± 0.04* | 0.60 ± 0.02 |

Results expressed as mg protein/well. Means ± 1 s.d. of triplicate assays. *$P < 0.01$ compared to controls. Put = putrescine.

**Figure 2** Effects of CsA and DFMO treatment on ODC activity in MOLT-4 cells in culture, measured by the release of $^{14}C$-$CO_2$ from $^{14}C$-ornithine. Results are means ± 1 s.d. of triplicate assays. ☐, Control; ▨, 2.5 $\mu$g ml$^{-1}$ CsA; ■, 5 $\mu$g ml$^{-1}$ CsA; ▧, 2.5 mM DFMO; ▩, 5 mM DFMO; *$P < 0.01$.

Figure 2 shows that ornithine decarboxylase (ODC) activity in the MOLT-4 cells was inhibited by treatment with DFMO (2.5 mM and 5 mM) as expected after 24 h and 48 h in culture. CsA (2.5 $\mu$g ml$^{-1}$ and 5 $\mu$g ml$^{-1}$) also lowered ODC activity after 24 h treatment with the higher concentration being the more effective. After 48 h in culture however, the ODC activity in the cells treated with 2.5 $\mu$g ml$^{-1}$ CsA appeared to have been fully restored to the same levels as the controls and the suppression of activity in the cells treated with 5 $\mu$g ml$^{-1}$ was markedly less than at 24 h.

### Discussion

We have investigated the effects of CsA on growth and polyamine metabolism of MOLT-4 cells in culture and compared the results to those obtained with the well established inhibitor of ODC, α-DFMO. Our data show that CsA inhibits cell growth in a dose-dependent manner demonstrated by the observed reductions in cell number, cell viability, protein content and $^3$H-TdR incorporation in cultures treated with a range of CsA concentrations.

Polyamines are known to be essential for cell growth and it has been suggested that depletion of polyamines was a potential mode of action for the growth inhibitory effects of CsA. However despite suggestions that polyamine biosynthesis may be involved in the antitumour action of CsA (reviewed, McLachlan *et al*., 1990), we observed no changes in intracellular polyamine content following addition of the drug to cultures. In contrast to previous reports (Saydjari *et al*., 1987) where the growth inhibitory effects of CsA could be overcome by the addition of putrescine, simultaneous addition of putrescine with CsA in our model did not reverse the effects of CsA on cell growth. We were however, able to reverse the actions of DFMO on intracellular polyamine content and cell growth by addition of putrescine. The lack of effect of CsA on intracellular polyamine levels is consistent with the results from our *in vivo* study (Smart *et al*., 1989), where we found that CsA did not deplete polyamine levels, nor did it enhance the polyamine depletion, seen with DFMO treatment, in blood mononuclear cells or in various tissue samples from the tumour hosts.

Despite the lack of a long term effect of CsA on polyamine content, CsA treatment did decrease ODC activity in MOLT-4 cells transiently (Table IV), suggesting that either CsA has a reversible effect on ODC or that there is a decreasing availability of CsA. Since CsA is known to bind to various molecules within the cell, such as cyclophilin (Merker & Handschumacher, 1984; Quesniaux *et al*., 1988; Ryffel, 1990) and the 170 Kd membrane P-glycoprotein which functions as a drug efflux pump in multidrug resistant cells (Foxwell *et al*., 1990; Nooter *et al*., 1990), decreased availability may be a major reason for the transient effect. DFMO is a 'suicide' inhibitor of ODC and binds irreversibly to the active site of the enzyme molecule (Metcalf *et al*., 1978) thus producing a direct effect on enzyme activity. On the other hand the reduction of ODC activity observed in CsA treated cells is more likely to be a consequence of the antiproliferative mechanism(s) of CsA on these cells.

It has been proposed by Sigal *et al*. (1990), that CsA inhibits $Ca^{2+}$-associated signal transduction pathways which may play a major role in the cascade leading to lymphokine production rather than directly inhibiting lymphokine mRNA transcription. These signals may also be linked to the rise in ODC activity which has been shown to be an integral event regluating lymphocyte DNA synthesis (Kay & Lindsay, 1973; Klimpel *et al*., 1979).

The intracellular binding protein cyclophilin is generally thought to be involved in the mode of action of CsA and has recently been shown to possess peptidyl-prolyl *cis-trans* isomerase (PPIase) activity which is inhibited by CsA binding (Takahashi *et al*., 1989; Fischer *et al*., 1989). Therefore it is possible that elements involved in the activation cascade may be 'conformationally' regulated by peptidyl-prolyl bond isomerisation. Alternatively, it may also be that intracellular metabolism of CsA may convert CsA to a derivative which has no effect on ODC.

Since CsA treatment does not affect intracellular polyamine content and its effects cannot be reversed by the addition of putrescine it seems more likely that the link between CsA effects and ODC activity is casual rather than specific and may exist for other enzymes whose functions, like that of ODC, are so closely linked to cell proliferation.

A recent report by Bowlin *et al*. (1989), concerning the effects of CsA and DFMO on the induction of cytolytic T lymphocytes *in vitro* and *in vivo*, demonstrated enhanced inhibition by combination of the drugs. The explanation proposed for this result was that the inhibition of interleukin-2 (IL-2) production by CsA was augmented by a decreased ability of polyamine-depleted cells to respond to IL-2. We hope that further studies on the effects of CsA, either on its own or in combination with DFMO, examining uptake and intracellular distribution of bound and free drug in different cellular compartments will help to clarify these observations and assist in design of possible therapeutic modalities.

We wish to thank the Merrell Dow Research Institute for the kind gift of DFMO, Sandoz Ltd for CsA and the Cancer Research Campaign for support in funding the project.

### References

BOREL, J.F., FEURER, C., MAGNEE, C. & STAHELIN, H. (1977). Effects of the new anti-lymphocyte peptide Cyclosporin A in the mouse. *Immunology*, **32**, 1017.

BOWLIN, T.L., ROSENBERGER, A.L. & McKOWN, B.J. (1989). α-Difluoromethylornithine, an inhibitor of polyamine biosynthesis, augments CsA inhibition of cytolytic T lymphocyte induction. *Clin. Exp. Immunol.*, **77**, 151.

FIDELUS, R.K. & LAUGHTER, A.H. (1986). Protein kinase activation and the immunosuppressant cyclosporine. *Transplanation*, **41**, 187.

FISCHER, G., WITTMANN, L.B., LANG, K., KIEFHABER, T. & SCHMID, F.X. (1989). Cyclophilin and peptidyl-prolyl *cis-trans* isomerase are probably identical proteins. *Nature*, **337**, 476.

FOA, P., MAIOLO, A.T., QUARTO DI PALO, F., STARACE, G. & POLLI, E. (1986). Cyclosporin A anti-leukaemia activity: mode of action on target cells. *Boll. Seroter. Milan*, **65**, 65.

FOXWELL, B.M.J., MACKIE, A., LING, E. & RYFFEL, B. (1990). Identification of the multidrug resistance-related P-glycoprotein as a cyclosporine binding protein. *Mol. Pharmacol.*, **36**, 543.

GRANELLI-PIPERNO, A., ANDRUS, L. & STEINMAN, R.M. (1986). Lymphokine and non-lymphokine mRNA levels in stimulated human T cells. Kinetics, mitogen requirements and effects of cyclosporine. *J. Exp. Med.*, **163**, 922.

HEBY, O. (1981). Role of polyamines in the control of cell proliferation and differentiation. *Differentiation*, **19**, 1.

HESS, A.D., TUTSCHKA, P.J. & SANTOS, G.W. (1982). Effect of CsA on human lymphocyte responses *in vitro*. *J. Immunol.*, **128**, 355.

KAY, J.E. & LINDSAY, V.J. (1973). Polyamine synthesis during lymphocyte activation. *Exp. Cell. Res.*, **77**, 428.

KINGSNORTH, A.N., LUMSDEN, A.B. & WALLACE, H.M. (1984*a*). Polyamines in colorectal cancer. *Br. J. Surg.*, **71**, 791.

KINGSNORTH, A.N., WALLACE, H.M., BUNDRED, N.J. & DIXON, J.M.J. (1984*b*). Polyamines in breast cancer. *Br. J. Surg.*, **71**, 352.

KLIMPEL, G.R., BYUS, C.V., RUSSEL, D.H. & LUCAS, D.O. (1979). Cyclic AMP-dependent protein kinase activation and the induction of ornithine decarboxylase during lymphocyte mitogenesis. *J. Immunol.*, **123**, 817.

LOWRY, O.H., ROSEBROUGH, N.J., FARR, A.L. & RANDALL, R.J. (1951). Protein measurement with the folin phenol reagent. *J. Biol. Chem.*, **193**, 265.

McLACHLAN, G., SMART, L.M., WALLACE, H.M. & THOMSON, A.W. (1990). The potential of cyclosporin A as an anti-tumour agent. *Int. J. Immunopharmac.*, **12**, 469.

MERKER, M. & HANDSCHUMACHER, R.E. (1984). Uptake and nature of the intracellular binding of cyclosporine A in a murine thymoma cell line BW5147. *J. Immunol.*, **132**, 3064.

METCALF, B.W., BEY, P., DANZIN, C., JUNG, M., CASARA, P. & VEVERT, J.P. (1978). Catalytic irreversible inhibition of mammalian ornithine decarboxylase by substrate and product analogues. *J. Am. Chem. Soc.*, **100**, 2551.

NOOTER, K., SONNEVELD, P., JANSSEN, A. & 6 others (1990). Expression of the mdr3 gene in prolymphocytic leukaemia: association with cyclosporin induced increase in drug accumulation. *Int. J. Cancer*, **45**, 626.

QUESNIAUX, V.F.J., SCHREIER, M.H., WENGER, R.M., HIESTAND, P.C., HARDING, M.W. & VAN REGENMORTEL, M.H.V. (1988). Molecular characteristics of cyclophilin-cyclosporin interaction. *Transplantation*, **46** (Suppl.), 23s–28s.

RUSSELL, D.H. & SYNDER, S.H. (1968). Amine synthesis in rapidly growing tissues: ornithine decarboxylase activity in regenerating rat liver, chick embryo, and various tumours. *Proc. Natl Acad. Sci. USA*, **60**, 1420.

RYFFEL, B. (1990). Pharmacology of cyclosporine. VI. Cellular activation: regulation of intracellular events by cyclosporine. *Pharmacological Reviews*, **41**, 407.

SAYDJARI, R., TOWNSEND, C.M. Jr, BARRANCO, S.C. & THOMPSON, J.C. (1987). Cyclosporine and α-difluoromethylornithine exhibit differential effects on colon and pancreatic cancer *in vitro*. *Invest. New. Drugs*, **5**, 251.

SIGAL, N.H., SIEKIERKA, J.J. & DUMONT, F.J. (1990). Commentary: observations on the mechanism of action of FK-506. *Biochem. Pharamcol.*, **40**, 2201.

SMART, L.M., McLACHLAN, G., WALLACE, H.M. & THOMSON, A.W. (1989). Influence of cyclosporin A and α-difluoromethylornithine, an inhibitor of polyamine biosynthesis, on two rodent T cell tumours. *Int. J. Cancer*, **44**, 1069.

TAKAHASHI, N., HAYANO, T. & SUZUKI, M. (1989). Peptidyl-prolyl *cis-trans* isomerase is the cyclosporin A binding protein cyclophilin. *Nature*, **337**, 473.

THOMSON, A.W., MOON, D.K., GECZY, C.L. & NELSON, D.S. (1983). Cyclosporine A inhibits the production of lymphokines but not the responses of macrophages to lymphokines. *Immunology*, **48**, 291.

THOMSON, A.W., FORREST, E.H., SMART, L.M., SEWELL, H.F., WHITING, P.H. & DAVIDSON, R.J.L. (1988). Influence of cyclosporine A on growth of an acute T cell leukaemia in PVG rats. *Int. J. Cancer*, **41**, 873.

TOTTERMAN, T.H., DANERSUND, A., NILSSON, K. & KILLANDER, A. (1982). Cyclosporin A is selectively cytotoxic to human leukaemic T cells *in vitro*. *Blood*, **59**, 1103.

TWENTYMAN, P.R. (1988). A possible role for cyclosporins in cancer research. *Anticancer Res.*, **8**, 985.

WALLACE, H.M. & KEIR, H.M. (1981). Uptake and excretion of polyamines from baby hamster kidney cells (BHK21/C13). The effect of serum on confluent cell cultures. *Biochim. Biophys. Acta*, **676**, 25.

WALLACE, H.M., GORDON, A.M., KEIR, H.M. & PEARSON, C.K. (1984). Activation of ADP-ribosyltransferase in polyamine depleted mammalian cells. *Biochem. J.*, **219**, 211.

WALLACE, H.M., NUTTALL, M.E. & ROBINSON, F.C. (1988). Actylation of spermidine and methylglyoxal bis(guanylhydrazone) in baby hamster kidney cells (BHK 21/C13). *Biochem. J.*, **253**, 223.